# Biosynthesis, Characterization, and Antifungal Activity of Novel Trimetallic Copper Oxide–Selenium–Zinc Oxide Nanoparticles against Some Mucorales Fungi

**DOI:** 10.3390/microorganisms11061380

**Published:** 2023-05-24

**Authors:** Amr H. Hashem, Abdulaziz A. Al-Askar, Józef Haponiuk, Kamel A. Abd-Elsalam, Mohamed S. Hasanin

**Affiliations:** 1 Botany and Microbiology Department, Faculty of Science, Al-Azhar University, Cairo 11884, Egypt; amr.hosny86@azhar.edu.eg; 2 Department of Botany and Microbiology, Faculty of Science, King Saud University, P.O. Box 2455, Riyadh 11451, Saudi Arabia; aalaskara@ksu.edu.sa; 3 Department of Polymer Technology, Faculty of Chemistry, Gdańsk University of Technology, G. Narutowicza 11/12, 80-233 Gdańsk, Poland; jozef.haponiuk@pg.edu.pl; 4 Plant Pathology Research Institute, Agricultural Research Center, Giza 12619, Egypt; 5 Cellulose and Paper Department, National Research Centre, El-Buhouth Street, Dokki 12622, Egypt

**Keywords:** trimetallic nanoparticles, biosynthesis, fungi, mucormycosis, antifungal activity

## Abstract

Metal nanoparticles are assumed to be a new generation of biologically active materials. The integrations between more than one metal are synergetic multifunctional features. In the current study, trimetallic copper–selenium–zinc oxide nanoparticles (Tri-CSZ NPs) were successfully mycosynthesized using *Aspergillus niger* through an ecofriendly method for the first time. The biosynthesis of the particles was characterized using physiochemical and topographical analysis. The physiochemical analysis included Fourier transform infrared spectroscopy (FTIR), which affirmed that the biosynthesis of Tri-CSZ NPs relies on the functional groups of fungal filtrates. Additionally, the UV–visible and X-ray diffraction patterns were proposed for the formation of Tri-CSZ NPs; moreover, topography analysis confirmed that the micromorphology of the nanoparticles were similar to a stick, with ends having a tetragonal pyramid shape, and with an average nanosize of about 26.3 ± 5.4 nm. Cytotoxicity results reveled that the Tri-CSZ NPs have no cytotoxicity on the human normal cell line Wi 38 at low concentrations, where the IC50 was 521 µg/mL. Furthermore, the antifungal activity of the Tri-CSZ NPs was evaluated. The antifungal results revealed that the Tri-CSZ NPs have promising antifungal activity against *Mucor racemosus*, *Rhizopus microsporus*, *Lichtheimia corymbifera*, and *Syncephalastrum racemosum*, where the minimum inhibitory concentrations (MICs) were 1.95, 7.81, 62.5, and 3.9 µg/mL, and the minimum fungicidal concentrations (MFCs) were 250, 62.5, 125, and 1000 µg/mL, respectively. In conclusion, Tri-CSZ NPs were successfully mycosynthesized using *A. niger*, which have a promising antifungal activity against fungi causing mucormycosis.

## 1. Introduction

Fungal infections have significantly increased in the last two decades, with high rates of mortality, especially in immunodeficiency patients [1]. Pathogenic fungi invade more than 1.2 billion individuals overall worldwide, with at least 1.7 million deaths/year [1,2]. The mortality of fungal pathogens becomes equal to drug-resistant *Mycobacterium tuberculosis* and exceeds malaria [3]. Besides aspergillosis and candidiasis, mucormycosis counts as the third most important disease in Europe in hematological patients [4]. Mucormycosis is considered to be a very destructive invasive fungal disease [5]. Human mucormycoses are caused by a wide range of fungal pathogens linked to the mucorales, including *Rhizopus*, *Mucor*, and *Lichtheimia corymbifera* and *Syncephalastrum* [6].

Mucormycosis is a fungus that often results from filamentous molds and is a member of the entomophthorales and mucorales orders [7]. Numerous habitats, such as soil, decomposing plant debris, bread, and dust, are where mucorales can be found [8]. Spore inhalation, ingesting contaminated food, or inoculating harmed surfaces or wounds are all ways to get a mucorales infection [9]. In a healthy, immunocompetent human, fungus spores that are present in the environment generally do not result in disease due to the body’s immune system. However, when the body’s defense system is weak, as it is in people with diabetes mellitus, neutropenia, organ transplant recipients, and other immune-compromised states, these fungal spores easily infiltrate our defense system, leading to a severe systemic infection, with roughly 45–80% of case fatalities [10,11]. Patients who were taking immunosuppressive medications, such as glucocorticoids, during the COVID-19 pandemic were at a significant risk of developing mucormycosis. Additionally, those with diabetes mellitus have a higher risk of contracting an infection. The spores typically enter our bodies through our respiratory system, where they harm the lungs and paranasal sinuses [12]. The widespread use of antifungal medications causes the development of fungi that are resistant to the majority of antifungal medications [13]. Recently, most pathogenic fungi have become resistant to drugs as well as bacteria. In order to control fungi that are resistant to medications, it is necessary to investigate or create novel antifungal drugs based on modern technology. 

According to many scientists, nanotechnology will be the next industrial revolution and will have a significant impact on society, the economy, and everyday life [14,15]. This field is highly beneficial in a number of areas, including agriculture, industry, wastewater treatment, and infection control [16,17,18,19,20]. The environmentally friendly method of biosynthesizing nanoparticles offers the potential for safe application in the medical domains [21,22]. Contrarily, biosynthesis techniques are increasingly becoming the most popular ones, since they are frequently one step, environmentally friendly, economical, secure, and clean, provided that the nanomaterial has more precisely defined sizes and morphologies [23,24,25]. Additionally, biological systems, which have been proposed as potential ecofriendly substitutes to physicochemical processes, operate as capping factors to stabilize NPs employing bacteria, fungi, and extracts of plant cells [26,27,28,29,30]. Since they have a high tolerance for metals and are simple to handle, fungi are desirable agents for the biogenic synthesis of silver nanoparticles. They also release a lot of extracellular proteins, which help the nanoparticles stay stable [31,32]. The benefits of fungal cultures over bacterial systems include superior biomass generation and the lack of additional extraction procedures. [33]. Fungi have shown great potential for the production of NPs on a large scale. Among the fungal sources, *Aspergillus* is a very promising candidate for the production of NPs because there are more than 350 species of this genus with enormous biochemical versatility, in addition to the secretion of a large quantity of proteins [34]. Different *Aspergillus* species produce NPs of diverse sizes and shapes with interesting physicochemical properties, such as enhanced thermostability, stability over a wide pH range, greater solubility, and biocompatibility. Moreover, the compounds produced by *Aspergillus* are classified with the generally regarded as safe (GRAS) status, which can be safely used in the industry [35]. Metal nanoparticles are also playing an important role in the process of overcoming the disadvantages of numerous fungal infections and fungal resistance [36,37]. In addition, the metal nanoparticles are presented as a pure metal or metal oxide [38]. Unfortunately, the antibacterial actions of a single nanometal do not affect all of the different types of germs, particularly those microbes that have developed resistance to the antimicrobial drugs. The requirement for new generations of antimicrobial agents that are relevant to microbial resistance can be supported by utilizing di- or trinanometal structures that are multifunctional and have multiple features that impact the microbial population [39]. Trimetallic nanoparticles are mainly formed by the combination of three different metals. The efficiency of the trimetallic catalysts is significantly higher than that of the bimetallic catalysts. The antibacterial activity of trimetallic nanoparticles is superior to that of bimetallic and monometallic nanoparticles according to a number of studies. One of the studies was the synthesis of gold, platinum, and silver by the use of a green approach, which involved the extraction of leaf material from Lamii albi flos. The acquired Au/Pt/Ag nanoparticles displayed exceptional antibacterial activities against hazardous strains of bacteria such as Enterococcus faecalis and Enterococcus faecium, which were both planktonic as well as sessile [40]. In this context, ZnONPs and CuONPs emerge a wide range of applications in the biomedical filed, which are used individually or bimetallically [41,42]. Additionally, selenium nanoparticles are used as an antimicrobial agent, with a nice cytocompatibility profile in comparison with the abovementioned nanoparticles [43]. The combination of the two or three metals or metal oxide in one structure provide multifeatured materials characterized with unique features and properties that are obtained from the integration of nanoparticles and which present a new feature related to the combination. For additional applications, nanoscientists and researchers have worked to optimize the size and/or morphology of these multimetallic NPs as heterodimers, core/shells, and alloys [44,45]. The literature describes the production of trimetallic NPs such as Sn-Zn-Cu [46], Pt60Ni3Cu37 [47], Au-Pt-Pd [48], and Au-Pt-Ag [49]. Herein, this study aims to (1) mycosynthesize novel trimetallic copper–selenium–zinc oxide nanoparticles using *Asperigillus niger* for the first time, (2) detect the safety of this novel compound through cytotoxicity against a human normal cell line, and (3) evaluate the antifungal activity against *Mucor racemosus*, *Rhizopus microspores*, *Lichtheimia corymbifera*, and *Syncephalastrum racemosum.*

## 2. Materials and Methods

### 2.1. Materials

All the chemicals, reagents, Potato dextrose agar (PDA) (product number, P2182) and Potato dextrose broth (PDB) (product number, P6685) media, aqueous copper acetate (product number, 341646), sodium selenite (product number, 71950), zinc acetate (product number, 1724703), and MTT stain (product number, 475989) were purchased from Sigma Aldrich (St. Louis, MO, USA). Human normal cell line Wi 38 was collected from the American type culture collection (ATCC). All the biological syntheses in this study were achieved using distilled water. 

### 2.2. Mycosynthesis of Tri-CSZ NPs

Tri-CSZ NPs were synthesized from *A. niger* AH1 biomass filtrate, which had previously been isolated, morphologically and genetically characterized, and deposited in a gene bank under the accession number MW680847 [50]. *A. niger* was inoculated into 250 mL Erlenmeyer flasks with 100 mL of Potato dextrose broth (PDB) medium. The inoculated flasks were incubated for 4 d, 150 rpm, at 28 °C 150 rpm. After removing the fungus’ mycelium from the culture medium using centrifugation (3500 RCF, 15 min, 4 °C), Whatman filter paper no. 1 was used for filtration to produce cell-free filtrate (CFF). Aqueous copper acetate, sodium selenite, and zinc acetate solutions were introduced in equal amounts to reaction vessels holding cell-free filtrate at a final concentration of 2.0 mM in order to create Tri-CSZ NPs. The reaction vessels were then incubated at 28 °C on a rotating shaker (150 rpm) without any light [51,52]. The presence of nanoparticles has been confirmed when the liquid turns a dark green color. The collected NPs were dried in an oven for overnight at 200 °C.

### 2.3. Characterizations

The characterizations of the Tri-CSZ NPs were carried out using physiochemical analysis including a diffraction pattern via XRD that was investigated using a Diano X-ray diffractometer (Philips, Amsterdam, The Netherlands) and UV–visible spectroscopy (V-630 UV–vis spectrophotometer (Jasco, Tokyo, Japan) in the range of 200–1000 nm) as well as an FTIR spectrometer (Nicolet Impact-400 FT-IR spectrophotometer) in the range of 400–4000 cm^−1^. Otherwise, the topographical study included a field emission SEM Model Quanta 250 FEG attached with energy-dispersive X-ray analysis; the Model Quanta 250 FEG (Field Emission Gun) attached with the EDX Unit (Energy Dispersive X-ray Analyses) for the EDX and the high-resolution transmission electron microscope (HRTEM) JEOL–JEM2010, Tokyo, Japan, was used to study the Tri-CSZ NPs particle shapes and sizes, as well as carried out the selected area diffraction (SEAD). 

### 2.4. In Vitro Cytotoxicity 

The cytotoxicity of the Tri-CSZ NPs was determined using the MTT protocol [53], with minor modification. Human normal cell line Wi 38 was collected from the American type culture collection (ATCC). The cell quantity and the percentage of viable cells were totaled by the following formula:

$$\mathrm{Viability}\;\%=\frac{\mathrm{OD}\;\mathrm{of}\;\mathrm{sample}}{\mathrm{OD}\;\mathrm{of}\;\mathrm{control}}\;\;\text{×}\;\;100$$

Inhibition %=100−Viability %

### 2.5. Antifungal Activity

Tri-CSZ NPs were tested for their antifungal efficacy against fungi causing mucormycosis, such as *Mucor racemosus* (accession no. MG547571.1) and *Rhizopus microsporus* (accession no. MK623262.1), *Lichtheimia corymbifera* (accession no. MK300698.1), and *Syncephalastrum racemosum* (accession no. MK621186.1). These fungal strains were cultured on Potato dextrose agar (PDA) plates, then incubated for 3–5 days at 28 ± 2 °C. Minor modifications were made to the well diffusion method used by Shubharani, et al. [54] to determine the antifungal activity of the Tri-CSZ NPs. Individually, each fungal strain was inoculated on PDB medium and then incubated at 28 ± 2 °C for 3–5 days. Individual fungal inoculums from each tested fungal strain were distributed on the surface of PDA plates. On each (90 mm) agar plate, 7 mm diameter holes were drilled with a sterile cork-borer. Then, 100 µL at a concentration of 2000 µg/mL was added to wells. The wells were individually filled with 100 µL of varied concentrations (0.98–2000 µg/mL) to determine the MIC. After 3–5 days of incubation at 25 °C, the zones of inhibition were observed and measured on the culture plates.

Radial growth was measured at various concentrations using the method developed by Joshi et al. [55], with minor modifications. The proportion of pathogen growth inhibition was estimated using the following equation:

$$\;\mathrm{Inhibition}\;\mathrm{of}\;\mathrm{pathogen}\;\mathrm{growth}\;\left(\%\right)=\frac{\mathrm{Growth}\;\mathrm{diameter}\;\left(\mathrm{control}\right)-\mathrm{Growth}\;\mathrm{diameter}\;\left(\mathrm{treatment}\right)}{\mathrm{Growth}\;\mathrm{diameter}\;\left(\mathrm{control}\right)}\times 100$$

## 3. Results and Discussion

### 3.1. Mycosynthesis of Tri-CSZ NPs

**Figure 1:**
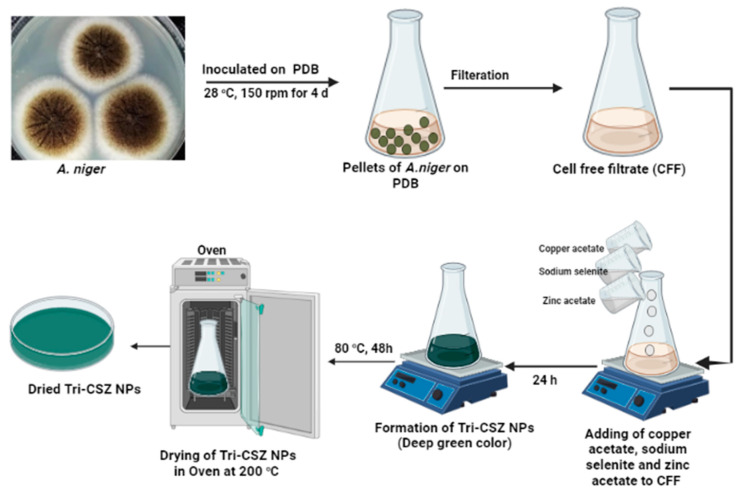
Biosynthesis of Tri-CSZ NPs using *A. niger.*

Fungi are easy, flexible, tolerant, and economic biologic systems for industrial biotechnology and have been used extensively in the high-scale production of different metabolites (primary and secondary) [56]. Tri-CSZ NPs were mycosynthesized using *A. niger* filtrate, which was used as a biocatalyst for the ecofriendly biosynthesis of these nanoparticles. A dark/deep green color was observed (Figure 1) after adding precursors (copper acetate, sodium selenite, and zinc acetate), which indicates the biosynthesis of the Tri-CSZ NPs and which is completely characterized in the following part. The biocatalyst with the necessary metabolic activity is the crucial stage in the process evaluation and determines the final output. Fungal metabolites may be responsible for the conversion of precursors to Tri-CSZ NPs via proteins, enzymes, and carbohydrates. Because of their ability to absorb and detoxify heavy metals via diverse reductase enzymes, microorganisms such as bacteria, fungi, viruses, and even yeast are also utilized in the manufacture of trimetallic nanoparticles [57]. Due to their strong tolerance for metals, particularly in light of the high biomass concentration of metal ions in cell walls, fungi have also been used to synthesize nanoparticles [14].

### 3.2. Trimetallic Nanoparticle Characterizations

Worthwhile, the Tri-CSZ NPs were characteristic using physiochemical and topographical analysis tools to confirm and understand the intermolecular and morphological structures and the arrangement of the formulation of Tri-CSZ NPs and features of particles, as well. 

### 3.3. Physiochemical Characterizations

**Figure 2:**
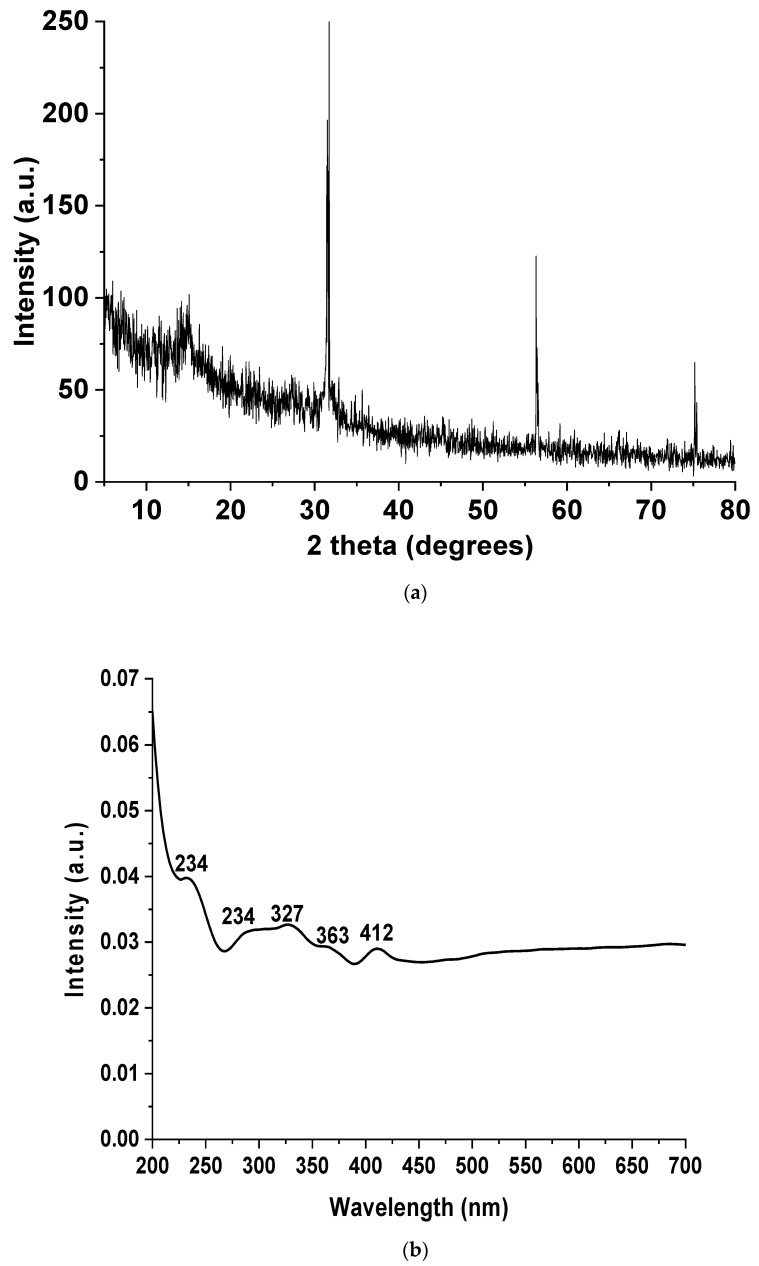
The diffraction pattern of the Tri-CSZ NPs (**a**) and UV–visible spectrum of the Tri-CSZ NPs (**b**).

The crystallographic pattern of the Tri-CSZ NPs is shown in Figure 2. The diffraction pattern clarified the presentation of all the metals as peaks at 14.5°, which relate to SeNPs, with shifts to a lower degree [58]. Furthermore, the peak at 31° was formed from the interaction of the three nanoparticle integrations with a high intensity [59,60]. Moreover, the peak at 35.7° was also related to the integration of ZnONPs and CuONPs [42]. Otherwise, the peaks at 56.3°, 66.5°, and 74° were due to ZnO, CuO, and Se nanoparticles, respectively. Consequently, the crystallographic diffraction pattern affirmed the introgression of the three metals together in nanoform with a hexagonal behavior, and these observations need to be confirmed with other spectroscopy investigations and emphasized with topographical analysis. 

Obviously, the UV–visible spectroscopy may provide useful information from this angle. The UV–visible spectrum of the Tri-CSZ NPs was illustrated in Figure 2b and confirmed the presence of the three nanometals without convention bands but in the range of peaks related to each metal. In particular, the bands at 230, 290, 327, 363, and 412 nm confirmed the presence of CuONPs, ZnONPs, and SeNPs, respectively, but without a specific recording because the bands overlap [42,61,62,63,64]. In this manner, the obtained results prospectively confirm the biosynthesis and integration of the trimetallic structure. 

**Figure 3:**
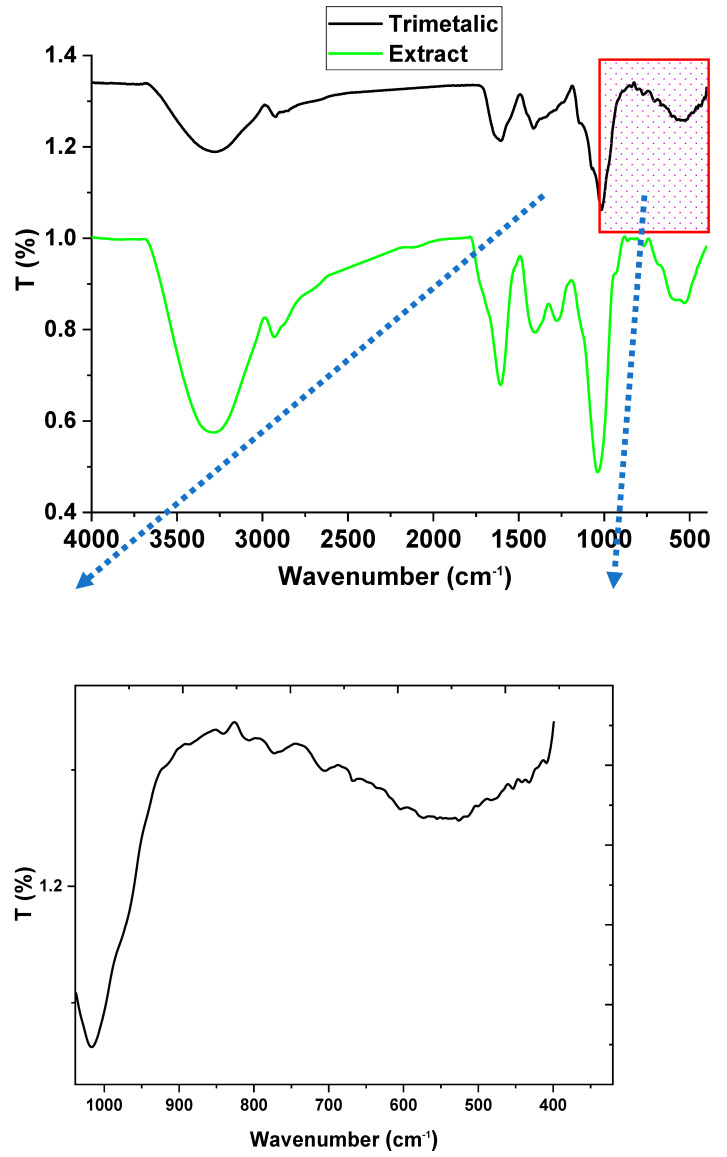
FTIR spectrum of fungal medium and Tri-CSZ NPs.

FTIR provide useful information about the biosynthesis efficiency and manipulation via tracking the functional groups changes. Figure 3 illustrates the FTIR spectrum of the fungal medium extract compared with the trimetallic spectrum. The fungal medium extract illustrated numerous functional groups that responded to the biosynthesis of the Tri-CSZ NPs, which presented the presence of hydroxyl groups and N-H groups as an overlapping of both functional groups at 3285 cm^−1^, which is the vibration stretching mode. The vibration stretching C-H bands were assigned as 2928 cm^−1^. Moreover, the bands at 1607, 1400, 1277, 1032, 771, and 589 cm^−1^ were attributed to C=O (amide I), C=O stretching and NH_2_ deformation, O-H in-plane deformation, C-O-C polysaccharide linkage, CH_2_ rocking, and OH out-plane bending, respectively [65]. In sum, in the functional groups of the fungal medium extract were observed the presence of active components working as caping and stabilizing agents for the formulation of nanoparticles. On the other hand, the spectrum of the Tri-CSZ NPs illustrates the functional groups involved in the formulation process of Tri-CSZ NPsin the range 4000 to 1000 cm^−1^. The hydroxyl group was shifted to 3295 cm^−1^ and the CH group band was split into two small groups at 2922 and 2832 cm^−1^. Moreover, the C=O band (around 1600 cm^−1^) intensity was decreased and the band at 1400 cm^−1^ was shifted to 1423 cm^−1^, while the polysaccharide band, CH_2_ rocking, and OH out-plane bending were shifted to lower frequencies. Herein, the FTIR spectrum of Tri-CSZ NPs in comparison with fungal filtrate spectrum was affirmed that the functional groups of the fungal filtrate significantly effect the biosynthesis of the nanoparticles. Additionally, the fingerprint region, which is less than 1000 cm^−1^, was observed in the bands relevant to the ZnO signal at 499 and 409 cm^−1^, with minor differences in comparison with the recordings in the literature [66,67] according to the integration between the trimetallic nanoparticle structures. Moreover, the CuO vibration bands were assigned at 619 and 515 cm^−1^ [68], while the SeNPs bands were recorded at 809 and 772 cm^−1^ [69]. Overall, the FTIR study obviously affirmed the role of the fungal medium extract in the formulation of Tri-CSZ NPs, as well as emphasized the evidence achieved by the XRD and UV–visible analyses. In this context, the physiochemical study using spectroscopy tools was confirmed and illustrated the formulation of the Tri-CSZ NPs.

### 3.4. Topographical Study

**Figure 4:**
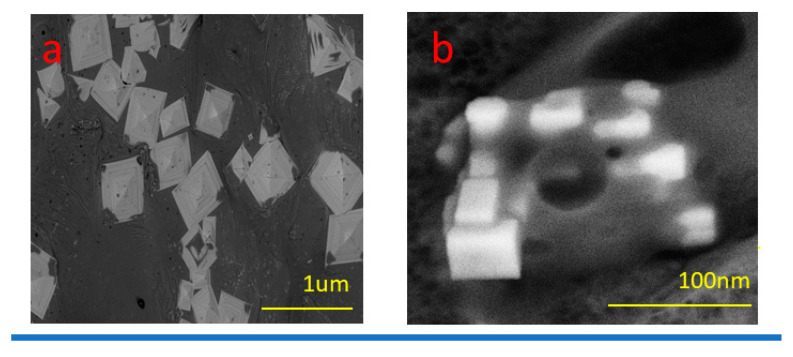

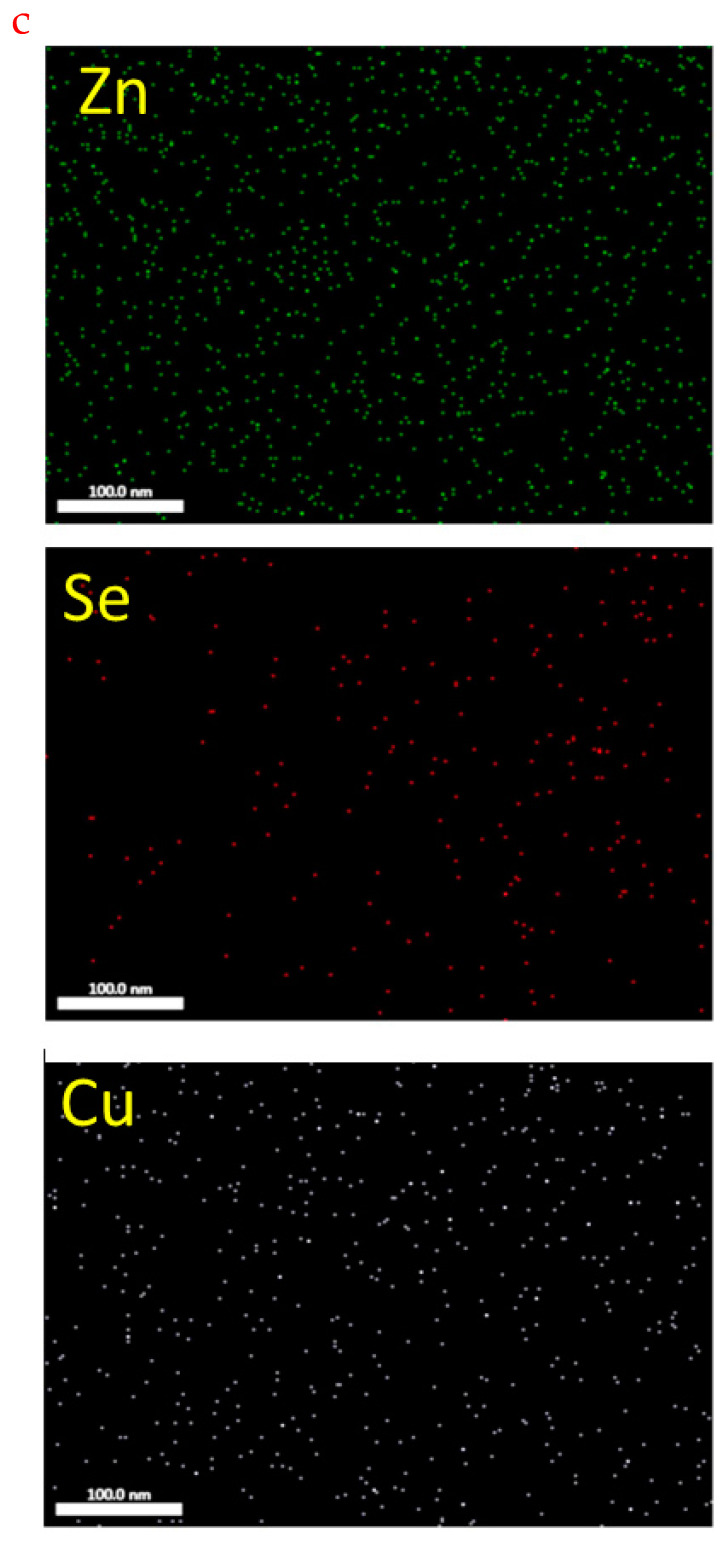

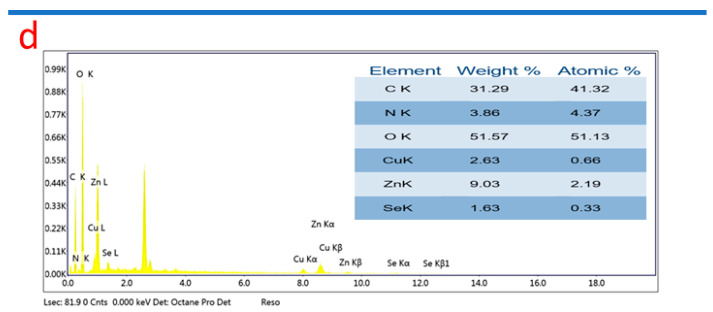
SEM images (**a**,**b**), mapping images of Zn, Se, and Cu atoms, (**c**) and EDX (**d**) of Tri-CSZ NPs.

The topography included the SEM coupled with the EDX and mapping charts, as well as the TEM coupled with the SEAD diffraction. The SEM images are shown in Figure 4 with a low magnification, which confirm that the metallic particles aggregated into a traditional morphological appearance of metal and metal oxide. The high magnification SEM image illustrated the tetragonal pyramid shape. This was not the only key feature, as the particle appeared as compact layers with clear borders and edges. This behavior could due to the different metals used in the biosynthesis. Moreover, the mapping confirmed the presence of Zn, Se, and Cu atoms, with a nice complementary distribution. In addition, the EDX chart clarified presence of carbon, and oxygen atoms were recorded in the highest amounts as shown in Figure 4d. This is attributed to the presence of metal oxide that captured a high amount of oxygen in comparison with nitrogen and carbon. Otherwise, the metal percentages were arranged from the highest percentage to the lowest, as Zn, Cu, and Se, respectively. These observations illustrate the Tri-CSZ NP morphologies and competent structures. These illustrations are in excellent agreement with the obtained conclusions from the physiochemical analysis. 

**Figure 5:**
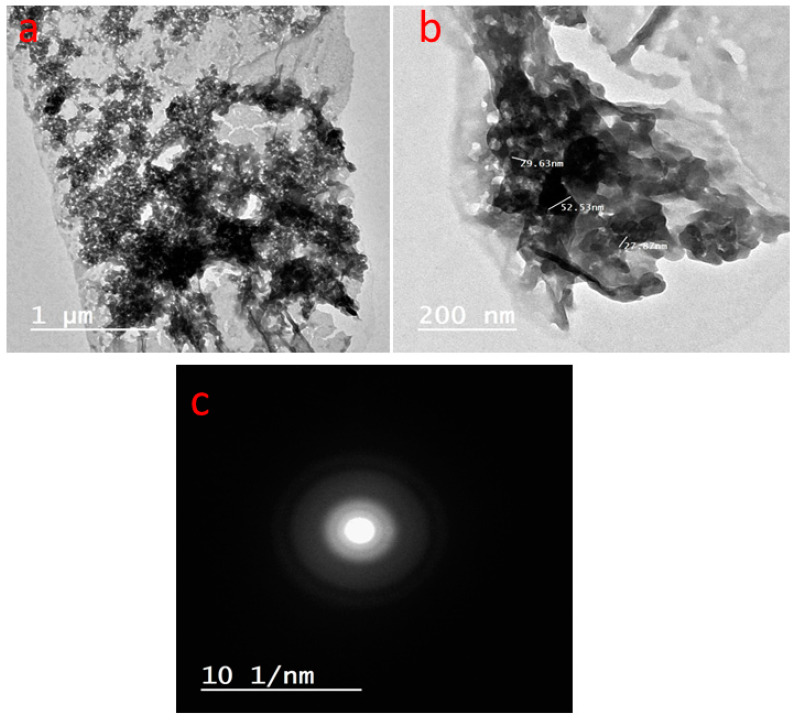
TEM images with low (**a**) and high (**b**) magnifications and SAED diffraction pattern of Tri-CSZ NPs (**c**).

On the other hand, the TEM images prove the particle shape and size, as shown in Figure 5. The appearance of the Tri-CSZ NPs was allocated as homogenous particles with an average size of around 26.3 ± 5.4 nm and a shape similar to a stick, with ends showing a tetragonal pyramid shape. These findings affirmed the SEM findings. Otherwise, the SEAD diffraction showed eight faint rings that reflected both side’s four faces (Figure 5c). Moreover, these rings were sharp and separated, which reflects a high crystallinity. 

### 3.5. Cytotoxicity

**Figure 6:**
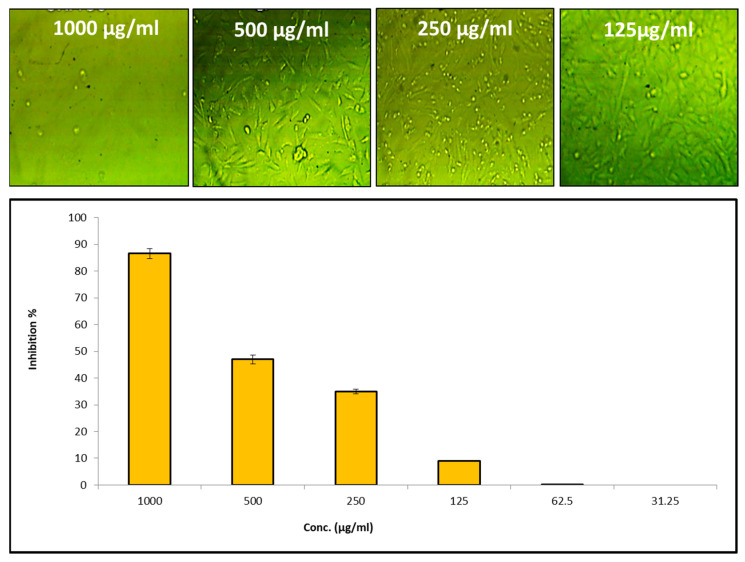
In-vitro cytotoxicity of Tri-CSZ NPs on human normal cell line Wi 38.

Cytotoxicity testing of the newly biosynthesized substances using in vitro testing of human normal cell lines is considered the initial step in determining whether or not these products are safe [70]. Figure 6 shows the results of an evaluation of the cytotoxicity of Tri-CSZ NPs using the Wi 38 normal cell line. According to the findings, the IC50 value for the Tri-CSZ NPs was 521 µg/mL. In general, the substance is considered to be noncytotoxic if the IC50 is less than 90 µg/mL [71]. Moreover, low concentrations of 62.5 and 31.25 µg/mL have no cytotoxicity on the Wi 38 normal cell line. This confirms that the prepared Tri-CSZ NPs are very safe if applied on live tissue.

### 3.6. Antifungal Activity

#### 3.6.1. Inhibition Zones and Minimum Inhibitory Concentrations

**Figure 7:**
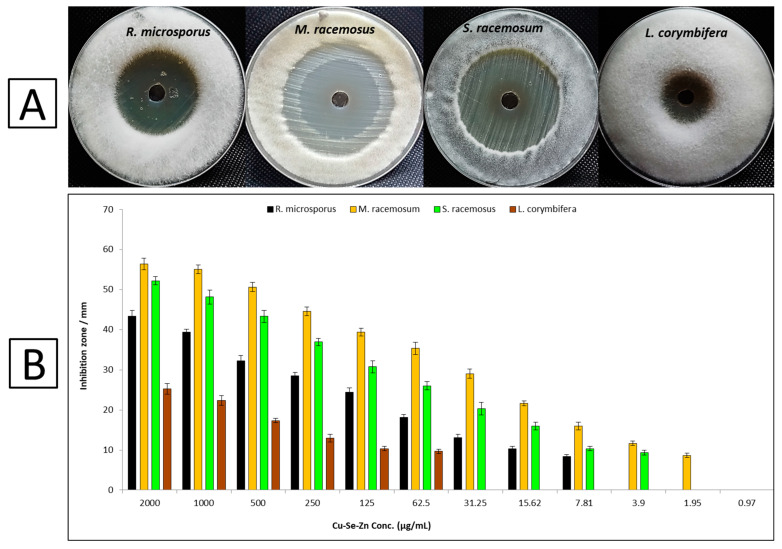
Antifungal activity of Tri-CSZ NPs against *R. microsporus*, *M. racemosus*, *S. racemosum*, and *L. corymbifera*: (**A**) Inhibition zones at a concentration of 2000 µg/mL; (**B**) Effect of different concentrations on the growth.

Silver nanoparticles (AgNPs), zirconium oxide nanoparticles (ZrO2NPs), and nanoemulsions (NB-201) are some of the latest antifungal medicines developed against various mucorales species. According to the fungal activity assays, silver nanoparticles enclosed in cyclodextrin can compete with mucorales, which inhibits the growth of *M. ramosissimus* [72]. Currently, there is still little knowledge on the connection between mucormycosis and the antifungal activities of nanoparticles. To combat the high mortality rates from mucormycosis in immunocompromised patients, the synthesis of novel antifungal medications is essential and urgent. In the current study, the antifungal activity of Tri-CSZ NPs was evaluated against fungi causing mucormycosis, such as *M. racemosus*, *R. microsporus*, *L. corymbifera*, and *S. racemosum*, as shown in Figure 7A. Results revealed that Tri-CSZ NPs showed promising antifungal efficacy against all fungal stains. Results also showed that M. racemosus is the most sensitive fungal strain tested, with an inhibition zone of 56 mm at a concentration of 2000 µg/mL. Moreover, it exhibited antifungal activity against *R. microsporus* and *S. racemosum*, but was lower against *M. racemosus*, where inhibition zones at 2000 µg/mL were 43 and 52 mm, respectively. On the other hand, *L. corymbifera* is the least sensitive among the other tested fungal strains (25 mm).

Furthermore, the MICs of the Tri-CSZ NPs against all tested fungal strains are shown in Figure 7B. Results revealed that the best MIC of the Tri-CSZ NPs was toward *M. racemosus,* where it was 1.95 µg/mL. Moreover, the MICs against *S. racemosum*, *R. microsporus*, and *L. corymbifera* were 3.9, 7.81, and 62.5 µg/mL, respectively. These concentrations of Tri-CSZ NPs are promising due to their effective antifungal activity, as well as having no cytotoxicity on normal cell lines at these concentrations.

The mechanisms of action of nanoparticles on fungi may be physical or chemical in nature. The size and surface properties of the nanoparticles have the biggest impact on the physical mechanism. The transit of electrons and ions, as well as the functioning of proteins, can all be affected by interactions between nanoparticles and biological systems, which can result in membrane disintegration [73]. Oxidative stress, nonhomeostasis, and coordination effects on fungi are further components of the chemical pathway. The production of reactive oxygen species (ROS) brought on by ZnO nanoparticles exceeds the fungal antioxidative limit, leading to fungal death [74]. Due to the disruption in the fungus’ natural redox state, the release of ROS damages fungal components such as lipids, proteins, and DNA [75]. Moreover, coordination effects damage DNA by transporting metal nanoparticles into nuclear pore complexes [76]. In addition, Zn^2+^ is a component of both proteins and enzymes that is essential for maintaining homeostasis; variations in Zn^2+^ levels can interfere with homeostatic systems. So, when ZnO nanoparticles release Zn^2+^ ions, the local content of metal ions goes up, which upsets the balance of metal ions in cells.

#### 3.6.2. Radial Growth and Minimum Fungicidal Concentrations

**Figure 8:**
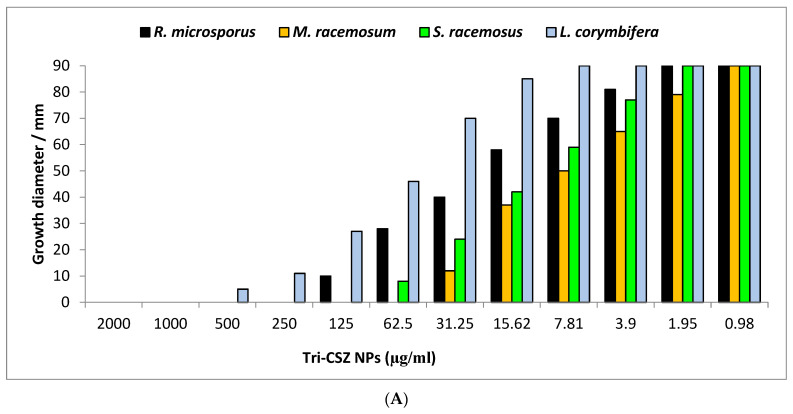

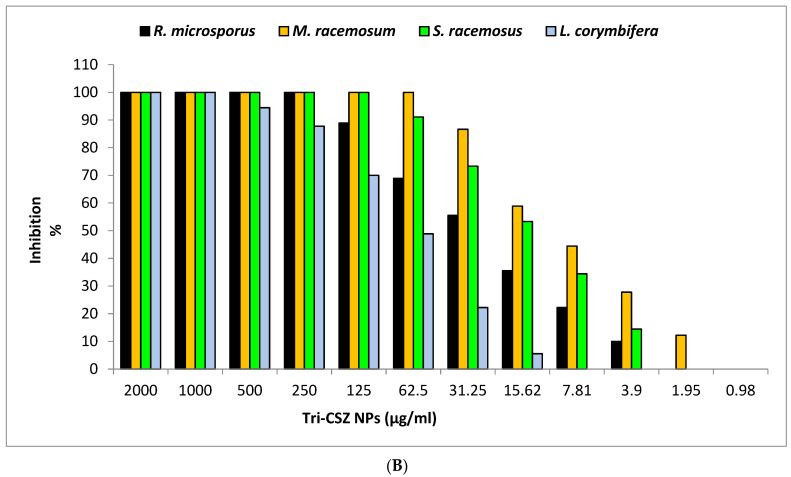
Radial growth (**A**) and inhibition percentages (**B**) of Tri-CSZ NPs at different concentrations against *R. microsporus*, *M. racemosus*, *S. racemosum*, and *L. corymbifera.*

The effects of different concentrations of Tri-CSZ NPs on the radial growth of *R. microsporus*, *M. racemosus*, *S. racemosum*, and *L. corymbifera* were evaluated, as shown in Figure 8. The radial growth of *R. microsporus*, *M. racemosus*, *S. racemosum*, and *L. corymbifera* was carried out to determine the growth inhibition percentage for each concentration of the Tri-CSZ NPs. Generally, the growth diameter increased with a decreasing concentration of nanoparticles, but the inhibition percentage decreased. Results illustrated that *M. racemosum* was the most sensitive among the other tested fungal strains, where the growth diameters were 90, 79, 65, 50, 37, 12, and 0 mm at different concentrations of 0.98, 1.95, 3.9, 7.81, 15.62, 31.25, and 62.5 µg/mL, respectively (Figure 8A), while the inhibition percentages at these concentrations were 0, 12.2, 27.8, 44.5, 58.9, 86.9, and 100%, respectively. On the other hand, *L. corymbifera* was the least sensitive among the others, where the growth diameters and inhibition percentages were 90, 85, 70, 46, 27, 11, 5, and 0 mm and 0, 5.5, 22.2, 48.9, 70, 87.8, 94.4, and 100% at concentrations of 7.81, 15.62, 62.5, 31.25, 62.5, 125, 250, 500, and 1000 µg/mL, respectively. Furthermore, the MFCs were determined for each test fungus, where the MFCs of the Tri-CSZ NPs toward *R. microsporus*, *M. racemosus*, *S. racemosum*, and *L. corymbifera* were 250, 62.5, 125, and 1000 µg/mL, respectively (Figure 8B).

## 4. Conclusions

In this study, Tri-CSZ NPs were mycosynthesized using *A. niger* for the first time. The produced Tri-CSZ NPs were characterized with unique features emphasized using physiochemical and morphological analysis. The Tri-CSZ NPs were estimated in the nano range with a high crystallinity, as well as with a homogeneous shape and distribution. Moreover, results illustrated that Tri-CSZ NPs are safe to use according to the cytotoxicity on the normal cell line Wi38. Furthermore, Tri-CSZ NPs had promising antifungal activity against *R. microsporus*, *M. racemosus*, *S. racemosum*, and *L. corymbifera*, which cause mucormycosis. Finally, the prepared mycosynthesized Tri-CSZ NPs, which have in vitro antifungal activity toward fungi causing mucormycosis, can be used after further in vivo studies.

## Data Availability

The data are made available upon request.
